# Docosahexaenoic acid has influence on action potentials and transient outward potassium currents of ventricular myocytes

**DOI:** 10.1186/1476-511X-9-39

**Published:** 2010-04-17

**Authors:** Ru-xing Wang, Xiao-rong Li, Tao Guo, Li-ping Sun, Su-xia Guo, Zhen-Yu Yang, Xiang-Jun Yang, Wen-ping Jiang

**Affiliations:** 1 Department of Cardiology, Affiliated Hospital of Nanjing Medical University in Wuxi and People's Hospital of Wuxi City, Wuxi 214023, China; 2 Department of Cardiology, First Affiliated Hospital of Kunming Medical College, Kunming 650032, China; 3 Department of Cardiology, First Affiliated Hospital of Soochow University, Suzhou 215006, China

## Abstract

**Background:**

There are many reports about the anti-arrhythmic effects of ω-3 polyunsaturated fatty acids, however, the mechanisms are still not completely delineated. The purpose of this study was to investigate the characteristics of action potentials and transient outward potassium currents (I_to_) of Sprague-Dawley rat ventricular myocytes and the effects of docosahexaenoic acid (DHA) on action potentials and I_to_.

**Methods:**

The calcium-tolerant rat ventricular myocytes were isolated by enzyme digestion. Action potentials and I_to _of epicardial, mid-cardial and endocardial ventricular myocytes were recorded by whole-cell patch clamp technique.

**Results:**

**1.** Action potential durations (APDs) were prolonged from epicardial to endocardial ventricular myocytes (*P *< 0.05). **2.** I_to _current densities were decreased from epicardial to endocardial ventricular myocytes, which were 59.50 ± 15.99 pA/pF, 29.15 ± 5.53 pA/pF, and 12.29 ± 3.62 pA/pF, respectively at +70 mV test potential (*P *< 0.05). **3.** APDs were gradually prolonged with the increase of DHA concentrations from 1 μmol/L to 100 μmol/L, however, APDs changes were not significant as DHA concentrations were in the range of 0 μmol/L to 1 μmol/L. **4.** I_to _currents were gradually reduced with the increase of DHA concentrations from 1 μmol/L to 100 μmol/L, and its half-inhibited concentration was 5.3 μmol/L. The results showed that there were regional differences in the distribution of action potentials and I_to _in rat epicardial, mid-cardial and endocardial ventricular myocytes. APDs were prolonged and I_to _current densities were gradually reduced with the increase of DHA concentrations.

**Conclusion:**

The anti-arrhythmia mechanisms of DHA are complex, however, the effects of DHA on action potentials and I_to _may be one of the important causes.

## Background

Fatty acids, especially polyunsaturated fatty acids (PUFAs), play an important role in life and death of cardiac cells. Reasons are as follow: **1.** they are essential fuels for mechanical, electrical, and synthetic activities of the heart; **2.** their levels are abnormally high in an ischemia followed by a reperfusion; and **3.** dietary fish oil is apparently beneficial for heart function [1,2]. Therefore, the beneficial effects of PUFAs on cardiovascular diseases, such as fish oil, have been reported, and the effects of PUFAs on anti-arrhythmias and prevention of sudden death have been highlights [3-6]. It has been reported that PUFAs have the roles of anti-arrhythmias and prevention of malignant ventricular arrhythmias, however, the mechanisms of which are still not completely delineated [7,8].

ω-3 PUFAs mainly include docosahexaenoic acid (DHA) and eicosapentaenoic acid (EPA). More attentions have been paid to their beneficial effects on cardiovascular diseases in recent years, especially in their anti-arrhythmias and prevention of sudden cardiac death [9-13]. However, most of these studies are clinical trials, and the mechanisms of which are still not completely known. To investigate the mechanisms of ω-3 PUFAs on anti-arrhythmias and prevention of sudden cardiac death [1,8], we observed the effects of DHA on action potentials and transient outward potassium currents (I_to_) of Sprague-Dawley rat ventricular myocytes by whole-cell patch clamp technique in this study. The results may provide some experimental evidences for rational applications of ω-3 PUFAs to prevent and treat arrhythmias in clinical practice.

## Methods

### Major experimental instruments

The instruments used were: MultiClamp 700B patch clamp amplifier (Axon Instruments, USA), D/A and A/D converter (DigiData 1322, Axon Instruments, USA), Pclamp 9.0 pulse software (Axon Instruments, USA), MP-285 motorized micromanipulator (Sutter Instruments, USA), IX71 inverted microscope (Olympus, Japan), SA-OLY/2 and DH-35 culture dish heater (Warner Instruments, USA), P-97 micropipette puller (Sutter Instruments, USA).

### Reagents, solutions and drugs

The reagents, solutions and drugs used were: DHA (Sigma, USA), molecular weight 328.5, 100 mmol/L stock solution was prepared by being dissolved in absolute ethanol and protected from light in refrigerator at -20°C. The experimental concentration of DHA was obtained by dilution of stock solution before each experiment. Action potential internal solution (in mmol/L) was KCl 120, CaCl_2 _1, MgCl_2 _5, Na_2 _ATP 5, EGTA 11, HEPES 10, glucose 11, pH 7.3 adjusted with KOH. Action potential external solution was Tyrode's solution [14]. I_to _external solution (in mmol/L) was NaCl 140, KCl 4, CaCl_2 _1.5, MgCl_2 _1, CdCl_2 _0.5, HEPES 5, glucose 10, pH 7.4 adjusted with NaOH. I_to _internal solution (in mmol/L) was KCl 140, MgCl_2 _1, K_2 _ATP 5, EGTA 5, HEPES 10, pH 7.4 adjusted with KOH. KB solution (in mmol/L) was L-Glutamic acid 50, KCl 40, KH_2_PO_4 _20, Taurine 20, MgCl_2 _3, KOH 70, EGTA 0.5, HEPES 10, glucose 10, pH 7.4 adjusted with KOH.

### Cell isolation

The investigation was approved by our institute ethics committee and conformed to the Guide for the Care and Use of Laboratory Animals published by the US National Institutes of Health (NIH publication No. 85-23, revised 1996). Healthy Sprague-Dawley rats of either sex, aged 8-12 weeks and weighing approximately 200 g, were provided by the Experimental Animal Center of Soochow University (Suzhou, China). Animals were anesthetized with pentobarbital sodium intraperitoneally (i.p.), Hearts were removed and retrograde perfusion through the aorta was performed as described [15]. After retrograde perfusion was finished, epicardial, mid-cardial, and endocardial ventricular myocardium were obtained respectively by cutting with eye scissor and plyer. Isolated cells were kept at room temperature in KB solution and used within 6 hr; only relaxed, striated, and rod-shaped cells were used.

### Recordings of action potentials and I_to _with and without DHA

Currents in whole-cell voltage clamp configuration were recorded following the method of Hamill et al [16]. Myocytes were transferred to a 1 ml chamber (DH-35 culture dish heater, Warner Instruments, USA) containing external solution placed on the stage of an inverted microscope. The chamber was continuously perfused at a rate of 1-2 ml/min with external solution. Electrodes were prepared from borosilicate glass (Clark Instruments, UK) using P-97 micropipette puller with resistances typically between 2 and 4 MΩ when filled with internal solution. Whole-cell voltage-clamp experiments were performed with MultiClamp 700B amplifier. Whole-cell capacitance and series resistance were compensated by 60-80%. Experiments were performed at 36-37°C. Voltage clamp pulses were generated via an IBM-compatible computer connected to Digidata 1322. Data acquisition and analyses were performed using pCLAMP software. To obtain action potentials, 5 ms depolarizing pulse with 900pA, 1 Hz in current-clamp configuration was applied. DHA at 0.01 μmol/L, 0.1 μmol/L, 1 μmol/L, 10 μmol/L, and 100 μmol/L was perfused for 10 min respectively to observe the influence on action potential durations (APDs). To obtain I_to_, 600 ms depolarizing pulses in the range -40 mV to +70 mV were applied to the ventricular myocytes every 5 s in +10 mV increment from -40 mV holding potential (HP). Recordings of action potentials and I_to _were performed at physiological temperature range (36-37°C). DHA at various concentrations was applied to investigate the effects on I_to_.

### Statistical analysis

Continuous variables were expressed as mean ± standard error ( ± *se*). SPSS11.5 (SPSS Inc, Chicago, Illinois, USA) was used for statistical analysis. Comparisons among groups were performed by repeated measurement analysis of variance (ANOVA) and least-significant difference contrast. Control and drug data for individual groups were compared by Paired *t*-test. *P *≤ 0.05 was considered significant. OriginPro 7.5 software (OriginLab, USA) was utilized to calculate the half-inhibited concentration (IC_50_).

## Results

### Characteristics of action potentials and I_to _of rat ventricular myocytes

**Figure 1:**
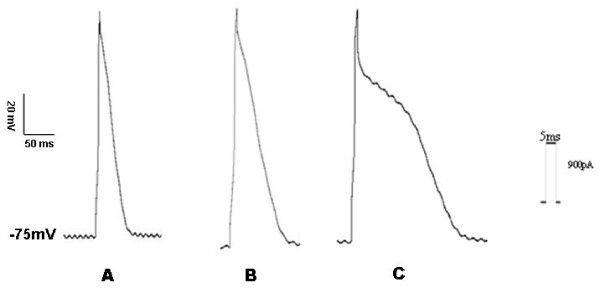
**Action potential morphologies of rat ventricular myocytes**: A, B and C were action potentials in epicardial, mid-cardial and endocardial ventricular myocytes, respectively. Action potential durations were gradually increased from epicardial ventricular myocytes to endocardial ventricular myocytes.

Action potentials of epicardial, mid-cardial and endocardial ventricular myocytes were recorded respectively with action potential stimulus protocol. The action potential morphologies were different in epicardial, mid-cardial and endocardial ventricular myocytes (Figure 1). APD_25_, APD_50_, and APD_90 _were gradually prolonged from epicardial to endocardial ventricular myocytes. Table 1 showed APDs' variations in myocytes of different layers; however, maximal velocity of action potential depolarization (Vmax), amplitude (APA), and overshoot (OS), didn't have remarkable changes in epicardial, mid-cardial and endocardial ventricular myocytes (Table 2).

**Table 1:** APD_25_, APD_50_, and APD_90 _of epicardial, mid-cardial and endocardial ventricular myocytes

	n	APD_25 _(ms)	APD_50 _(ms)	APD_90 _(ms)
Epi	50	3.6 ± 1.2	10.3 ± 2.1	46.3 ± 4.8
Mid	58	6.4 ± 1.8	14.7 ± 2.4	69.4 ± 8.3
Endo	62	13.8 ± 2.1	45.3 ± 10.2	152.1 ± 33.4

*P *value		*P *< 0.05	*P *< 0.05	*P *< 0.05

Epi, Mid, and Endo represent epicardial, mid-cardial and endocardial ventricular myocytes respectively

**Table 2:** Vmax, APA, and OS of epicardial, mid-cardial and endocardial ventricular myocytes

	n	Vmax (V/s)	APA (mV)	OS (mV)
Epi	71	228.3 ± 14.5	110.7 ± 10.1	31.5 ± 5.4
Mid	63	231.2 ± 13.4	111.9 ± 9.3	32.4 ± 6.3
Endo	70	226.9 ± 12.8	109.8 ± 8.9	30.8 ± 4.8

*P *value		*P *> 0.05	*P *> 0.05	> 0.05

Epi, Mid, and Endo represent epicardial, mid-cardial and endocardial ventricular myocytes respectively, and Vmax, APA, and OS represent maximal velocity of action potential depolarization, action potential amplitude and overshoot

**Figure 2:**
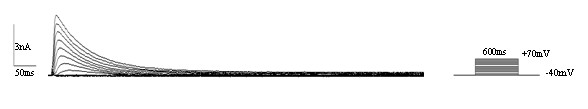
**The representative current tracings of transient outward potassium currents at various test potentials**: Currents were elicited by 600 ms depolarizing in the range of -40 mV to +70 mV pulses in +10 mV increments from -40 mV holding potential. The currents were enhanced with the increase of test potentials, activated rapidly and inactivated slowly.

I_to _current tracings at various test potentials were elicited by 600 ms depolarization in the range of -40 mV to +70 mV pulses applied to the ventricular myocytes every 5 s in +10 mV increments from -40 mV HP. The representative current tracings were shown in figure 2. The current densities of epicardial, mid-cardial and endocardial ventricular myocytes at +70 mV were 59.50 ± 15.99 pA/pF, 29.15 ± 5.53 pA/pF, and 12.29 ± 3.62 pA/pF, respectively.

**Figure 3:**
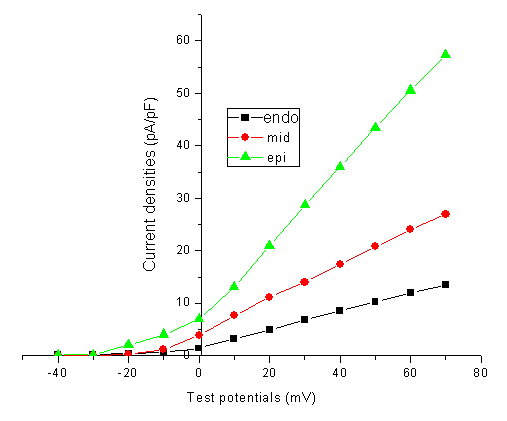
**The current-voltage curves of transient outward potassium currents in epicardial, mid-cardial and endocardial ventricular myocytes**: Currents in epicardial, mid-cardial and endocardial ventricular myocytes were all enhanced with the increase of test potentials from -40 mV to +70 mV, whereas currents in epicardial were higher than those in mid-cardial and endocardial at the same test potential, and currents in mid-cardial were larger than those in endocardial.

The current-voltage curves of I_to _were plotted with current densities at each test potential (Figure 3). The threshold potential of I_to _channel opening was -30.3 ± 2.8 mV, i.e., I_to _channel began to activate at more than -30 mV. I_to _currents were gradually enhanced with the increase of test potentials. The activation of I_to _channel was very rapid, and only needed to about 10 ms, nonetheless, its inactivation was relatively slow. The time constants of epicardial, mid-cardial, and endocardial ventricular myocytes were almost the same at each test potential. They were 31.8 ± 1.7 ms, 32.9 ± 2.4 ms, and 33.2 ± 2.9 ms, respectively, at +70 mV test potential (*P *> 0.05).

### Effects of DHA on action potentials

**Figure 4:**
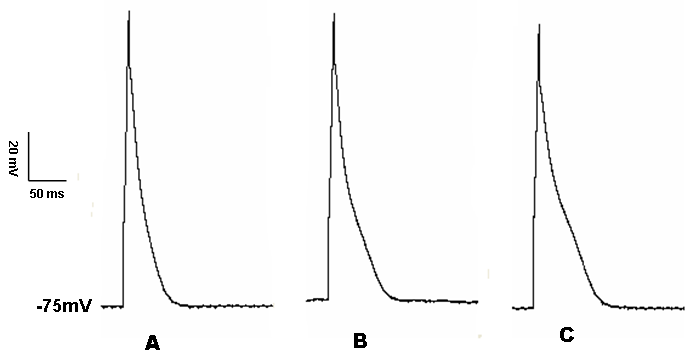
**Action potential changes of rat ventricular myocytes at 0.1 μmol/L DHA**: A, B, and C were morphologies of action potential when 0.1 μmol/L DHA was applied at 0 min, 1 min, and 5 min, respectively. The action potential duration was increased; however, compared with the control (0 min), the prolongation of action potential duration was less than 15%.

**Figure 5:**
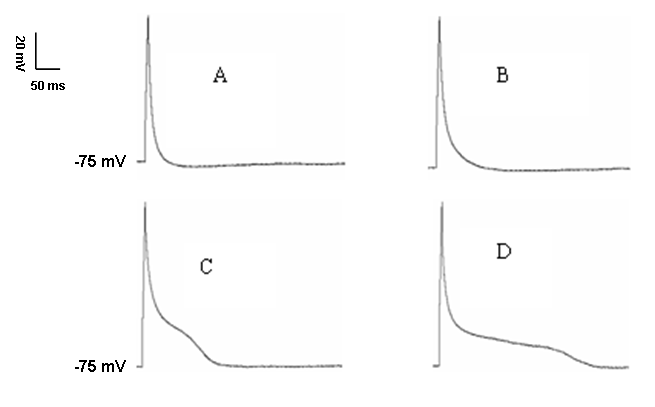
**Action potential changes of rat ventricular myocytes at different DHA concentrations**: A, B, and C were the applications of 10 μmol/L DHA at 0 min, 1 min, and 5 min. D was DHA at concentration of 100 μmol/L for 5 min. Action potential durations were significantly prolonged in concentration-dependent manner when DHA concentrations were more than 10 μmol/L.

DHA at 0.01 μmol/L, 0.1 μmol/L, 1 μmol/L, 10 μmol/L, and 100 μmol/L was applied on epicardial ventricular myocytes, respectively. The results showed that: ① APDs were gradually prolonged with the increase of DHA, whereas APDs' changes were not significant at low concentration of DHA (<1 μmol/L). The prolongation of APD_25_, APD_50_, and APD_90 _was less than 15% compared with the control (0 min) when 0.1 μmol/L DHA was applied (Figure 4). ② APDs were prolonged in a concentration-dependent manner when DHA concentrations were more than 1 μmol/L. APD_25_, APD_50_, and APD_90 _were 7.7 ± 2.0 ms, 21.2 ± 3.5 ms, and 100.1 ± 9.8 ms respectively when 10 μmol/L DHA was used at 5 min (Figure 5C). APD_25_, APD_50_, and APD_90 _were 15.2 ± 4.0 ms, 45.7 ± 6.8 ms, and 215.6 ± 15.7 ms respectively when 100 μmol/L DHA was utilized at 5 min (Figure 5D), which was significantly prolonged compared with those without addition of DHA (*P *< 0.05).

### Effects of DHA on I_to_

**Figure 6:**
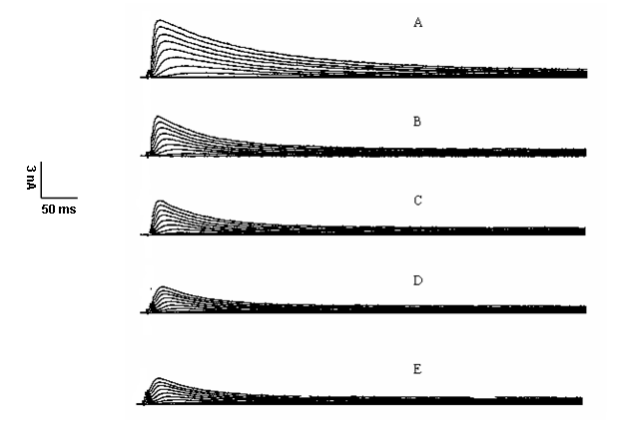
**Alterations of transient outward potassium currents after DHA at 100 μmol/L was applied**: A, B, C, D, and E were representative transient outward potassium current tracings with DHA at 0 min, 1 min, 5 min, 10 min, and 15 min, respectively. Transient outward potassium currents were remarkably blocked by DHA.

DHA at 0.01 μmol/L, 0.1 μmol/L, 1 μmol/L, 10 μmol/L, and 100 μmol/L was applied, respectively. I_to _currents were blocked by DHA in a concentration-dependent manner. Currents were gradually decreased with the increase of DHA concentrations. The current density of I_to _at +70 mV was 30.1 ± 7.2 pA/pF with DHA at 100 μmol/L. The representative current tracings blocked by DHA at 100 μmol/L were shown in figures 6. IC_50 _of DHA on I_to _was fitted with Hill function and calculated by OriginPro 7.5 software, which was 5.3 μmol/L.

## Discussion

The typical action potential consists of 5 phases or stages, i.e., 0, 1, 2, 3, and 4. The present study has showed that the rat ventricular myocytes don't have the typical action potential morphology. APDs are the shortest in epicardial ventricular myocytes, and repolarization rapidly appears after depolarization, showing no platform phase [17]. The phenomenon of "spike and dome" [18] sometimes can be seen, however, this phenomenon does not appear in endocardial ventricular myocytes. Action potential repolarization in endocardial ventricular myocytes is slow, showing relative standard action potential morphology. The action potential morphologies of mid-cardial ventricular myocytes are between epicardial and endocardial ventricular myocytes [19,20], but action potential repolarization is still rapid, and has the tendency of platform phase compared with the epicardial ventricular myocytes. The reasons why these alterations appear are that there are regional differences of I_to _in epicardial, mid-cardial and endocardial ventricular myocytes. The I_to _channels are the most abundant in rat epicardial ventricular myocytes and then in mid-cardial and endocardial ventricular myocytes by turns. The I_to _current densities in epicardial, mid-cardial and endocardial ventricular myocytes were different in this study, which further illustrates that I_to _channels of rat ventricular myocytes have regional differences. I_to _channels in epicardial myocytes are extremely abundant, and therefore, I_to _currents are the largest, which makes action potential repolarization rapid, calcium inflow time and APDs short. In contrast, I_to _channels in endocardial myocytes are few or lack, and therefore, APDs prolong. APA, Vmax, and OS are formed mainly by 0 phase depolarization. Because regional differences of I_to _channels do not affect depolarization of ventricular myocytes, and thus, APA, Vmax, and OS don't have significant differences in epicardial, mid-cardial and endocardial myocytes [21].

The Ca^2+^-insensitive but 4-aminopyridine-sensitive I_to _currents play a major role in modulating cardiac electrical activity [22]. It underlies phase 1 repolarization, and thus, by setting the voltage of the early plateau phase, it influences activation and inactivation of other plateau currents that control repolarization. It has also been reported in several studies that I_to _channels are potentially important targets for both neuromodulatory control [23] and antiarrhythmic drug actions [24]. These currents have been suggested to contribute significantly to the regional electrophysiological heterogeneity within the ventricular wall, a fact considered to be responsible for T-wave polarity. The heterogeneous distribution of I_to _thus appears to be essential in causing the transmural electrical gradients necessary for proper repolarization of cardiac action potentials. It is expected that changes in I_to _distribution and availability can be expressed in the ECG by typical J-wave and T-wave alterations and may lead to cardiac arrhythmias during evolving heart diseases.

The present study results have showed that there are regional differences of action potentials and I_to _amplitude and morphology of rat epicardial, mid-cardial and endocardial myocytes. Regional differences of action potentials and I_to _should be considered when rats are chosen as the experimental animal. We should try to obtain same regional ventricular myocytes to avoid experiment errors when we perform cellular electrophysiological study. Consequently, in this study, we only chose epicardial myocytes to investigate the effects of DHA on action potentials and I_to_.

ω-3 PUFAs mainly include DHA and EPA. In order to investigate the mechanisms of ω-3 PUFAs on anti-arrhythmias and prevention of sudden death, we performed this experiment to study the effects of DHA on action potential and I_to _of rat ventricular myocytes. The reasons that we chose the rats as experimental animal is not only because the rats have many advantages, e.g. cheap, strong vitality, and easily bred, but more important is that there are many similar electrophysiological characteristics of rat and the human being's cardiomyocytes [25,26].

After DHA at various concentrations was applied, APDs were gradually prolonged and I_to _currents were decreased by degrees with the increase of DHA concentrations. DHA could inhibit I_to _currents, prolong APDs, and extend effective refractory period of myocardial myocytes [27]. The effects of DHA on action potentials and I_to _may be one of its anti-arrhythmia mechanisms.

Figure 6 clearly showed that DHA could inhibit I_to _currents. However, from the morphologic changes of action potentials in figure 5, we found that the morphologic changes of action potentials mainly appeared in phase 2 and phase 3 with the increase of DHA concentrations. In contrast, action potential phase 1 formed mainly by I_to _current efflux didn't have significant changes. This means DHA may have the effects on other ion currents in addition to I_to _current.

The present study has some limitations, e.g., we only investigated the effects of DHA on action potentials and I_to _of rat ventricular myocytes. On the other hand, ω-3 PUFAs not only include DHA but also EPA, the electrophysiological effects of EPA on rat ventricular myocytes, and DHA on other ion currents such as I_Na_, I_Ca-L_, I_K_, and I_K1 _are still needed to further study. Only we completely explore the anti-arrhythmia mechanisms of ω-3 PUFAs, we can apply them correctly in clinical practice to prevent and treat cardiovascular diseases [28-32].

## Conclusion

In summary, the present findings obtained by the patch-clamp technique have clearly shown that APDs are prolonged, and I_to _currents are gradually reduced with the increase of DHA concentrations. Effects of DHA on action potentials and I_to _may be one of the important causes of anti-arrhythmias.

## Competing interests

The authors declare that they have no competing interests.

## Authors' contributions

XRL, XJY, and WPP designed research and revised the manuscript; RXW, LPS, SXG, and ZYY performed research; RXW wrote the manuscript; RXW and TG analyzed data. All authors read and approved the final manuscript.
